# Future North Atlantic tropical cyclone intensities in modified historical environments

**DOI:** 10.1038/s41597-025-06186-z

**Published:** 2025-12-05

**Authors:** Nicholas Lalo, Wenwei Xu, Lili Yao, Ning Sun, Karthik Balaguru, Julian Rice, Serena Lipari, Travis Thurber, Zhaoqing Yang, Mithun Deb, David Judi

**Affiliations:** Pacific Northwest National Laboratory, Richland, WA USA

**Keywords:** Projection and prediction, Atmospheric dynamics

## Abstract

Tropical cyclones (TCs) have ranked as the deadliest and most financially crippling natural disasters in the United States. It is imperative to assess potential shifts in TC intensity within the paradigm of an evolving climate. In this study we apply a fixed-constraint storyline approach that holds storm tracks and initial conditions constant to probe future TC intensity in the North Atlantic Basin. First, we simulate 618 historical TC events using the Risk Analysis Framework for Tropical Cyclones (RAFT)’s deep-learning intensity model. Next, we apply warming signals derived from eight CMIP6 climate scenarios and rerun each event to explore how intensities respond across scenarios. Finally, we develop an interactive dashboard that allows users to explore individual storm simulations and the scenario-modified environmental drivers. Together, this dataset and tool provide a clear, illustrative way to investigate how TC intensity responds to changes in air-sea state.

## Background & Summary

**Fig. 1:**
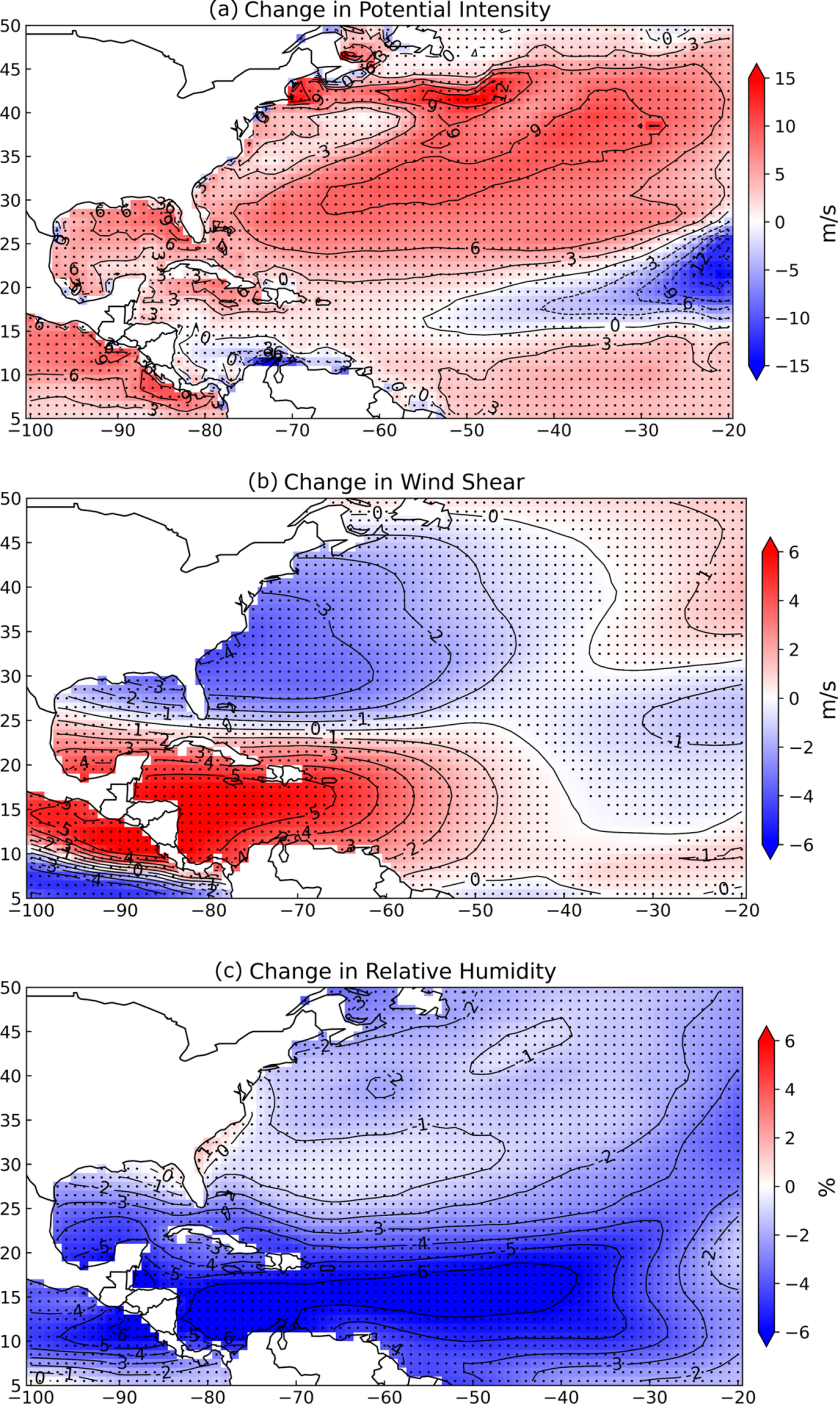
The difference in 40-year hurricane season mean between SSP585 far-future hot model (2059-2098) and historical (1979-2018) values, calculated using monthly ensemble averages from 8 GCMs. Black dots in each plot indicate grids with significant changes at the 95% confidence level using the Student’s t-test. The data show key environmental factors that influence TC intensification: (**a**) potential intensity, computed using sea surface temperature, mean sea-level pressure, specific humidity, and air temperature; (**b**) 200-850 hPa vertical wind shear; and (**c**) 700-850 hPa relative humidity.

Given that an estimated 85% of damages in the North Atlantic Basin are caused by major hurricanes (Category 3 and above)^1^, understanding future tropical cyclone (TC) intensities, particularly for major hurricanes, is critical for safeguarding public safety and economic stability. While there is significant uncertainty in how the frequency of North Atlantic TCs may change^2^, a significant upward trend in the proportion of major hurricanes has been observed over the past four decades^3^, along with increases in both the lifetime maximum intensity and intensification rates of the strongest storms^2,4–6^. Projections from the Coupled Model Intercomparison Project Phase 6 (CMIP6) suggest substantial changes in the environmental conditions that drive TC development^7^. In our fixed-constraint storyline experiment, where we hold storm tracks and initial conditions constant, we utilize ensemble averages of monthly data from eight General Circulation Models (GCMs) within CMIP6, spanning the period from 1979 to 2100, grouped by their climate sensitivity into high-sensitivity (“hot model”) and low-sensitivity (“cold model”) categories, depending on the strength of their response to warming signals. Figure 1 illustrates the projected changes in key environmental factors under the Shared Socioeconomic Pathway (SSP) 585 scenario, for the hot model in the far future period (2059-2098), compared to historical values (1979-2018). These factors include potential intensity, vertical wind shear, and relative humidity. Notably, potential intensity, which indicates the maximum storm intensity that environmental conditions can support, is projected to increase over much of the North Atlantic Basin. However, in the main development region (10 to 20°N and 20 to 60°W), the average VMPI actually decreases slightly by 0.16 percent. At the same time, vertical wind shear, a factor that typically suppresses TC intensity by disrupting air circulation, is projected to weaken near the US coast. Relative humidity is also projected to decline both in the main development region and across much of the basin. Although Fig. 1 does not show air temperature change, our calculations at the 1000 hPa level indicate a mean warming of 4.75°C over the North Atlantic Basin under these SSP585 late-century conditions. Together, these shifts in thermodynamic and dynamical drivers outline the input changes to our future reruns of historical storms.

Numerous studies have addressed future changes in TC activity by simulating storms in both historical and future climates. Focusing on the North Atlantic Basin, Knutson *et al*. (2008) used a regional climate model to explore changes in TC frequency^8^, while Murakami *et al*. (2010) examined shifts in TC tracks using a global atmospheric model^9^. Murakami *et al*. (2011, 2012) expanded their research to assess TC activity in the Western North Pacific^10^ and later conducted a global analysis^11^. In work more closely related to the research presented here, Bhatia *et al*. (2018) investigated global-scale TC intensification projected through the year 2100 using a coupled atmosphere-ocean GCM whose SSTs were relaxed toward projected SST anomalies^12^. Patricola *et al*. (2018) analyzed a subset of storms in historical climates and compared them to those modified by prehistoric and future climates^13^. Building on this approach, Jones *et al*. (2023) applied thermodynamic climate change signals to historical weather events using dynamical downscaling, allowing for a physics-based analysis of how extreme events might evolve under future climate conditions^14^.

Jones *et al*. provide a unique set of CMIP6-derived climate projections of past observed events at a 12-km resolution over the continental U.S. Their approach dynamically downscales a 40-year historical weather sequence from 1980 to 2019, driven by European Center for Medium-Range Weather Forecast’s reanalysis v5 (ERA5) climate reanalysis data, and repeats this sequence with the addition of eight distinct thermodynamic warming signals corresponding to four 80-year future warming trajectories from 2020 to 2099. These trajectories, based on CMIP6 climate scenarios SSP585 and SSP245, and derived from GCMs with varying climate sensitivities, offer a valuable framework for assessing climate risks and exploring adaptive responses. The use of a consistent baseline across all projections allows for straightforward comparison of future changes, providing insights into how thermodynamic shifts could reshape the characteristics of historical extreme weather events, including TCs. However, the WRF model, like other atmospheric models, encounters difficulties in accurately representing TCs above Category 3^15^. Additionally, the geographical constraints of their WRF simulation limit the analysis of North Atlantic Basin TCs to the Gulf and the U.S. East Coast.

Adopting a method inspired by the work of Jones *et al*.^14^, our study utilizes a simplified version of their thermodynamic warming signal approach. We employ the Risk Analysis Framework for Tropical Cyclones (RAFT)’s intensity model to simulate historical TC events and then rerun them with the addition of warming signals from eight future climate scenarios. RAFT is an established framework that integrates statistics, deep learning, and physics-based models to create synthetic representations of TC tracks, intensities, and associated rainfall^16–18^. A key distinction between our method in this work and that of Jones *et al*.^14^ lies in the modeling approach: while the previous study utilizes the WRF regional atmospheric model (WRF-TGW), applying future warming signals to the boundary conditions, RAFT’s intensity model leverages deep learning to directly incorporate future warming signals into the model’s inputs from observed initial conditions and along predetermined historical storm tracks. Unlike dynamical WRF-TGW runs, which explicitly solve the governing equations at high resolution, RAFT’s data-driven framework bypasses those computational demands by learning statistical relationships—albeit at the cost of relying on patterns present in the training data rather than first-principles physics. RAFT’s deep learning model is known for its ability to simulate intense storms above Category 3^16^. This enables the RAFT dataset to complement WRF-TGW by more effectively simulating major TCs, particularly Category 3 and 4 storms, given that both models utilize the same future climate scenarios. Additionally, to overcome WRF-TGW’s geographical limitations, RAFT’s modeling domain includes the full North Atlantic Basin allowing for the simulation of these intense storms across their full lifecycles. By integrating deep learning insights across the full North Atlantic Basin, this method offers a scenario-based view of how TCs might evolve along their historical tracks when exposed to different warming signals, complementing the coastal focus of WRF-TGW’s simulations.

**Fig. 2:**
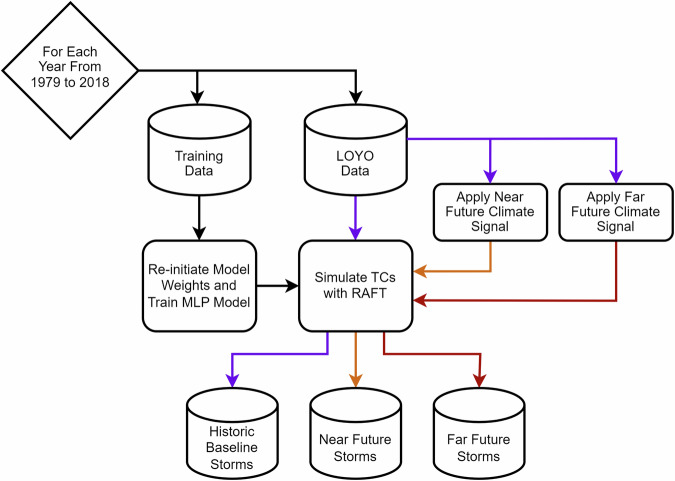
The workflow for training Multi-Layer Perceptron (MLP) models and simulating TC intensities in the North Atlantic Basin. A Leave One Year Out (LOYO) method is applied across a 40-year period, where each year’s TCs are simulated using a model trained on data from all other years. This approach maximizes training data while preventing data leakage. Shown in purple, RAFT uses each TC’s initial intensity, track data, and historical environmental data to simulate TC intensities along the entire track. In the future warming simulations, shown in orange (Near Future) and red (Far Future), warming signals are applied to historical environmental data to project how these storms would behave in the context of future climates.

In summary, we leverage the deep learning intensity model within RAFT to generate a dataset of 618 historical TCs spanning from 1979 to 2018, as shown in Fig. 2. We then apply eight distinct future warming signals, covering a wide but plausible range of warming scenarios, to re-simulate these events for two future periods: a near-future period from 2019 to 2058 and a far-future period from 2059 to 2098. The main contributions of this work are:

1. RAFT’s deep learning intensity model simulates historical and future collections of TCs across the North Atlantic Basin, providing insights into potential intensification patterns of future storms under a range of warming scenarios.
2. To facilitate broader access and engagement, we have developed a web-based dashboard that allows for interactive exploration of the simulated storm data, enabling users to visualize the changing intensities of individual storms across both historical and projected future scenarios.

## Methods

### Model Training

RAFT’s intensity model^16^, a deep learning Multi-Layer Perceptron (MLP), is trained to predict 6-hourly intensity changes (either 10m maximum sustained 1-minute wind speed or the instantaneous maximum wind speeds) using global data from the Statistical Hurricane Intensity Prediction Scheme (SHIPS) dataset^19^. The model’s input features, listed in Table 1, are primarily sourced from the SHIPS dataset, with the exception of LP500_t0, which represents the land percentage within 500 km of the storm center. During training, the model receives SHIPS data at each step in a storm and is trained to predict the 6-hour intensity change for the next step. Given that the model only takes in 10 variables and is trained on a dataset totaling 75,000 storm steps, a grid search is used to find the optimal model architecture and hyperparameters. To maximize the number of North Atlantic Basin TCs in the training data while preventing data leakage, a Leave-One-Year-Out (LOYO) cross-validation method is employed, as depicted in Fig. 2. For example, to simulate storms in the North Atlantic Basin in the year 2005, a model is trained on data from all other TC basins except the North Atlantic Basin in the year 2005. This ensures the model has no prior knowledge of 2005 storms or their environment. The LOYO method is applied for every year in the 40-year range, creating 40 marginally different models. Model performance is evaluated using the left-out data from each year as validation. This validation data is given to the model in the same way as the training data, with SHIPS data provided at each step, and 6-hourly intensity changes are predicted.

**Table 1:** Variables used in the RAFT dataset NetCDF4 files.

Variable Name	Model Input	Description
vmax_kts	Yes	Simulated instantaneous maximum wind speed in knots
VMPI_t0	Yes	Maximum potential intensity from Kerry Emanuel equation in knots
U200_t0	Yes	200 hPa zonal wind within 200 to 800 km at current position in knots*10
SHRD_t0	Yes	850 minus 200 hPa shear within 200 to 800 km at current position in knots*10
DELV_6	Yes	Last 6-hourly intensity change in knots
LP500_t0	Yes	Land percentage within 500 km of current position
lat_t0	Yes	Current storm center latitude in degrees North
lon_t0	Yes	Current storm center longitude in degrees East (negative for West of the Prime Meridian)
PSLV_v3	Yes	Zonal transport speed in 6 hours in m/s * 10
RHLO_t0	Yes	Relative Humidity at current position in percentage
dvs6	No	Predicted 6-hourly intensity change in knots
DTL_t0	No	Distance to major landmass from current position in kilometers
IBTrACS_vmax	No	Observed instantaneous maximum wind speed in knots from the IBTrACS dataset
datetime_strings	No	Datetime string for the storm steps from the historical period
year	No	Year the storm started
storm_names	No	Name of storm if named, otherwise Not_Named
storm_ID	No	Index of storm in corresponding IBTrACS data file

### Historical Baseline

A 40-year simulation of historical storms in the North Atlantic Basin is conducted using observed TC tracks and initial intensities from the International Best Track Archive for Climate Stewardship (IBTrACS) dataset^20^, along with environmental inputs from the SHIPS dataset and ERA5^21^. For each step, SHIPS data is used for environmental inputs if the IBTrACS latitude and longitude match a SHIPS storm track within 0.1 degrees; otherwise, ERA5 reanalysis data is used. The model predicts a 6-hour intensity change, which is added to the initial intensity and propagated forward for the next step. This autoregressive process continues until the end of the historical storm track or until the storm’s intensity falls below 10 knots. Additionally, because our focus is on the North Atlantic Basin, we end the simulation of storms which travel into the Pacific Ocean. Two other modifications to the original intensity model are Maximum Potential Intensity (VMPI) decay and forced survival. VMPI decay applies a smooth 10% reduction per 6-hour step over land, where VMPI is undefined. Forced survival ensures that the first four 6-hourly steps of a storm’s simulation are predicted as no change or positive intensity change. After completing the simulation, the model’s performance is evaluated by comparing simulated intensities to 6-hourly IBTrACS intensities. For any step where RAFT ends the storm early, the simulation assigns an intensity of zero.

### Warming Signals

Future change signals from eight GCMs were calculated for five variables: air temperature, relative humidity, northward wind speed, eastward wind speed, and sea surface temperature. Monthly data from these GCMs, with 21 ensemble members in both SSP585 and SSP245 scenarios, were utilized to calculate the future change signal. While this signal contains both dynamical and thermodynamic components, we refer to it as a warming signal to match the language from Jones *et al*.^14^. Within the pool of GCMs, four models exhibited greater sensitivity to warming, characterized by higher transient climate response (TCR) and equilibrium climate sensitivity (ECS) values, while the remaining four were less sensitive, with lower TCR and ECS values. These metrics quantify how much the global mean temperature responds to increased greenhouse gas concentrations. The distinction between “hot” and “cold” models reflects the models’ ability to simulate relatively stronger or weaker warming responses, with the hot models projecting more pronounced future temperature increases. These subsets are referred to as the “hot model” and “cold model” groups, respectively, throughout this manuscript. The selection of GCMs, ensemble members, and methods for calculating warming signals adhered to the approach outlined by Jones *et al*.^14^. Historical data spanning from 1975 to 2014 and future projections from 2015 to 2100 were extracted from each ensemble and standardized to a common 1-degree latitude-longitude grid. For each variable, ensemble averages were computed, followed by model averages for both the cold and hot model groups. To enhance data smoothness, a moving average with an 11-year window centered on each year was calculated for each year between 1979 and 2098. For example, the average for January 2020 is the moving average of January from 2015 to 2025. The moving average values consist of three 40-year segments, including the historical segment (1979-2018), near-future segment (2019-2058), and far-future segment (2059-2098). Monthly deltas were then derived for each year in 2019-2058 and in 2059-2098 relative to the corresponding years in 1979-2018.

Maximum Potential Intensity, VMPI in the model inputs, serves as an indicator of the maximum intensity a storm can attain at a specific location from thermodynamic considerations^22^. The computation of VMPI involves essential input parameters, including sea surface temperature, mean sea-level pressure, and the entire vertical profile, 1000 hPa to 1 hPa, of both specific humidity and air temperature provided by the GCMs. Therefore, the former four variables from each GCM ensemble were also used, and the moving average of each variable from 1979 to 2098 was determined through a consistent methodology, aligning with the approach used for the computation of other variables as described in previous paragraphs. The tcpyPI Python package, based on the^23^ algorithm with reversible adiabatic ascent assumed, is employed to calculate the VMPI values^24,25^ and the monthly deltas of VMPI are subsequently derived for each year in the near future (2019-2058) and far future (2059-2098) relative to the corresponding years in the historical period (1979-2018). Figure 1 shows the difference in 40-year hurricane season mean between SSP585 far future hot model and historical values.

Using the calculated warming signals as environmental deltas from the eight future scenarios, RAFT’s intensity model reruns historical storms in the context of future environments as seen in Fig. 2. In these future simulations, each TC is initialized in the same way as in the historical baseline simulation, with the same track and initial intensity. However, at each time step, deltas are applied to the environmental variables, including vertical wind shear, u200 wind component, VMPI, and relative humidity. As in the baseline simulation, the model predicts 6-hourly intensity changes autoregressively until the storm track ends or the intensity drops below 10 knots.

#### Overview of Future TC Intensity Results

Following the simulation of historical events from 1979 to 2018, the model inputs are modified by projected warming signals for the near- and far-future, enabling the re-simulation of historical TCs under eight different climate scenarios. We hold initial conditions, storm tracks, and overall TC frequency constant and employ a forced survival criterion that prohibits any simulated decrease in intensity during the first 24 hours. This setup enables us to assess how a fixed set of historical storms responds to modified environments rather than representing a comprehensive projection of future TC intensity.

We evaluate the distributions of intensity, lifetime maximum intensity, landfall intensity, and intensification rate for each scenario, depicted in Fig. 6. Figure 6(a) and 6(b), showing instantaneous and lifetime maximum wind speeds, reveal marginal increases in both 75th- and 99th-percentile intensities for SSP245 and SSP585 near-future scenarios, while the SSP585 far-future displays a modest decrease. Landfall intensities (Fig. 6(c)) show slight decreases in median values across all future scenarios, with minimal changes in extreme landfall events. Lastly, Fig. 6(d) illustrates minimal changes in rapid-intensification and weakening events across all but the SSP585 far-future scenarios, which feature fewer occurrences of both.

These trends must be interpreted within the context of this study’s fixed-track storyline framework, which forces historical storms to persist through modified environments for at least 24 hours rather than dynamically resolving storm formation or frequency changes. Direct comparisons to probabilistic projections, such as those reported in Knutson *et al*.^2^, are problematic due to significant methodological differences. In probabilistic studies, weaker storms may fail to develop or survive in future climates, potentially increasing the relative proportion of intense storms. In contrast, our approach retains weaker storms, leading to conservative intensity estimates.

Despite these limitations, the regional trends mapped in Fig. 8 provide valuable insights into storm-environment interactions. Across most scenarios, ensemble-mean intensity declines in the central Gulf of Mexico, while the U.S. East Coast shows slight intensity increases. Notably, the Caribbean Sea exhibits a pronounced drop in simulated wind speeds ( ~10-15 kt in several grid cells) under SSP585 far-future cold and hot models (panels f and h). This regional variability underscores how competing climate signals - such as changes in VMPI, shear, and moisture - balance within modified environments.

Finally, while this study focuses on the RAFT intensity model as part of a controlled experimental design, complementary research employing the full RAFT multi-model framework has explored bias-corrected synthetic storms in more freely evolving simulations to examine future changes in tropical cyclone frequency, intensity, and rainfall^18,26,27^.

## Data Records

The historical and future simulated TC dataset generated by RAFT’s intensity model is publicly accessible at 10.57931/2588708^28^. The dataset includes 618 reproduced historical TCs from 1979 to 2018, along with projections of these storms under future environmental conditions corresponding to eight future warming scenarios. The top-level directory contains an example script view_historic_and_future_Irene_example.py and a subfolder named RAFT_data. Inside RAFT_data is the IBTrACS input file IBTrACS_NA.v04r00.nc, which supplies initial intensities and track data for the experiment, the historical baseline run RAFT_historic_baseline.nc, and eight future-scenario NetCDF4 files. The future simulations all follow the naming convention RAFT_ssp <scenario>_<futurePeriod>_< modelMean>.nc. Each RAFT file contains the NetCDF variables listed in Table 1. In each NetCDF file, aside from the variables storm_names, year, and storm_ID, all data fields are stored as two-dimensional arrays (storm  × time step), where the first dimension indexes the storm tracks and the second corresponds to the IBTrACS recorded track. RAFT simulates storms at 6-hourly steps, whereas the IBTrACS dataset provides data at 3-hourly steps, with additional entries for significant events such as landfall. To reconcile this discrepancy, RAFT adds masked steps between the simulated 6-hourly steps to account for the extra IBTrACS data points. To assist users in utilizing the dataset, the example script shows how to parse the data with and without masked steps as well as generate a simple visualization of the simulated and observed vmax_kts.

### Interactive Dashboard for Simulation Exploration

To help users explore both historical recreations and projected future changes to TCs, an interactive web-based dashboard is available at RAFT-hurricane-projections.msdlive.org (see Fig. 7). This platform features an intensity line plot and a map, allowing users to filter or display all available simulations by clicking on the check boxes for various simulations above the line plot. The dashboard also includes a detailed “more” section at the bottom, offering insights into the model inputs and how they are affected by future signals. After finding a storm of interest with the search bar, the IBTrACS observed and RAFT historical intensities are shown with the option of clicking on check boxes above to add or remove more simulations from the line plot. This tool is intended as a quick and easy way for potential users to investigate the dataset without the need to download and parse the data themselves.

## Technical Validation

### Historical and Future Simulation Validation

**Fig. 3:**
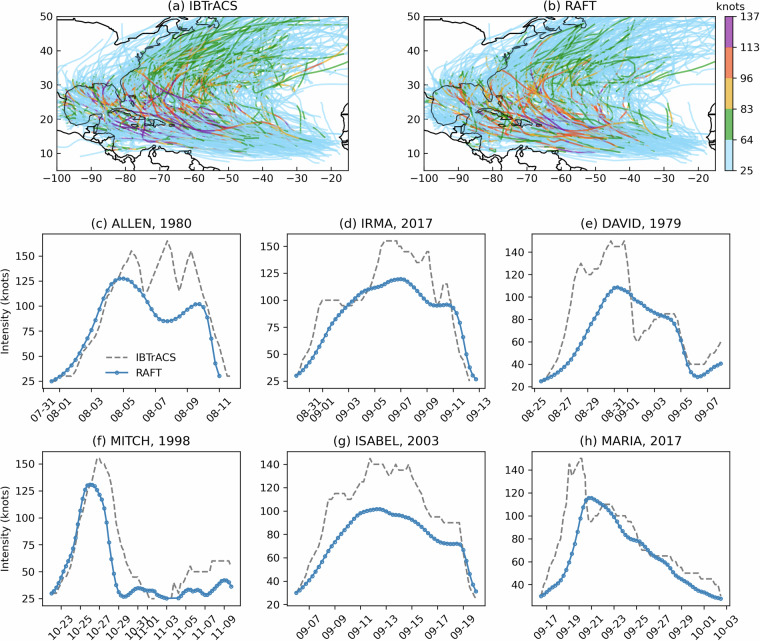
Comparison of 618 TCs in the North Atlantic Basin between 1979 and 2018 from the IBTrACS dataset (**a**) and RAFT’s historical simulations (**b**). Panels (**c**)-(**h**) show the six TCs in the North Atlantic Basin sustaining Category 5 strength for the longest duration in the historical 40-year time period simulated in this dataset.

**Fig. 4:**
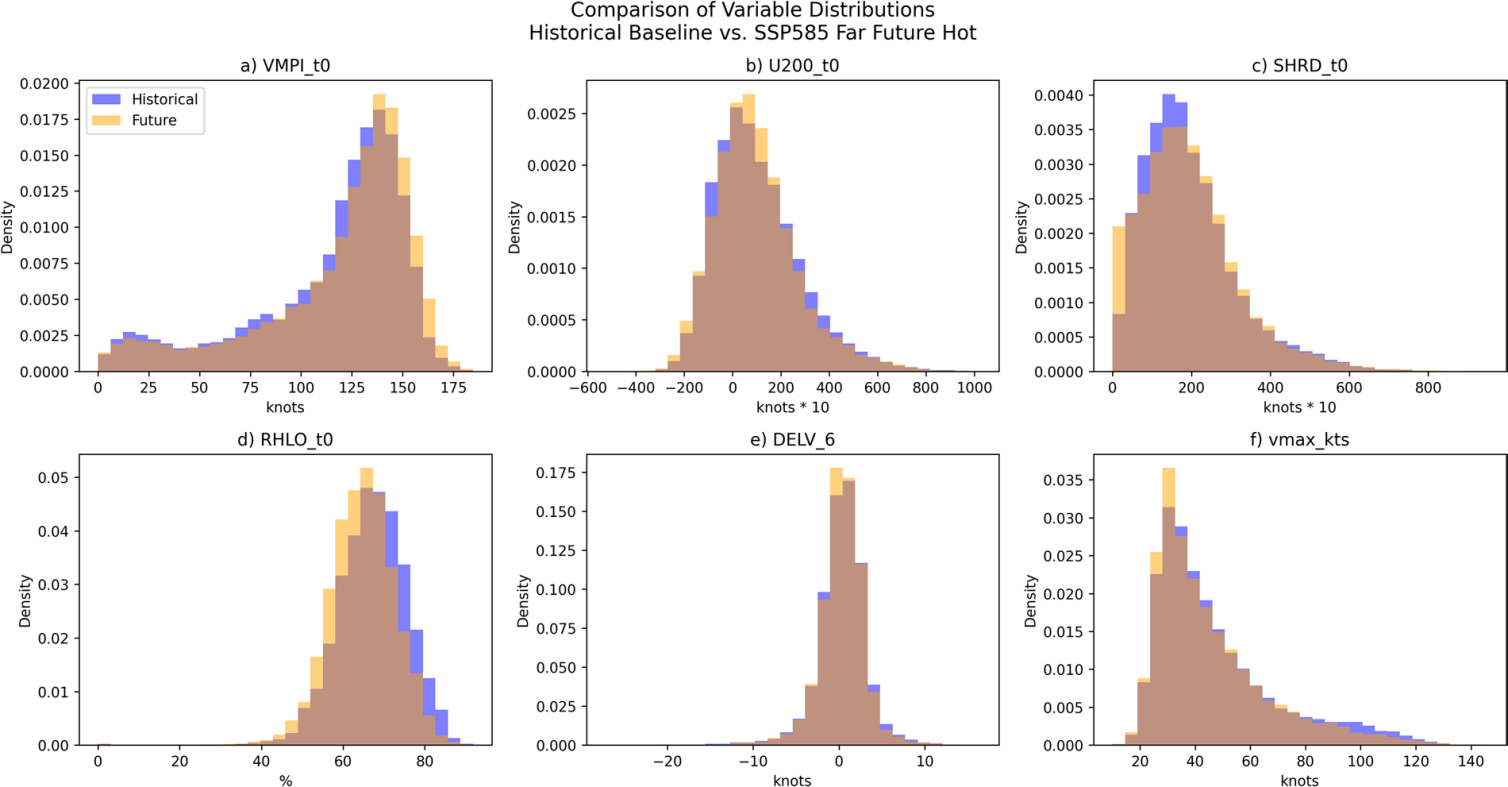
Distributions of key model inputs under the historical baseline and the SSP585 far-future hot scenario (the case with the largest environmental shifts). Panels (**a**-**d**) show the four warming-signal-adjusted environmental variables, and panels (**e**-**f**) show the previous 6-hour intensity change and current intensity.

To assess the validity of the TCs generated by RAFT’s intensity model, we begin by analyzing the simulations that recreate historical storms. Figure 3(a),(b) compare the spatial distribution of all storms from 1979 to 2018 in the IBTrACS dataset with RAFT’s simulated storms. This broad comparison demonstrates RAFT’s ability to realistically represent the spatial distribution of TCs. However, a noticeable discrepancy occurs north of 30 degrees latitude where RAFT tends to under predict TC intensity. More detailed validations of RAFT’s intensity model’s performance is presented in Lipari *et al*.^26^, and Rice *et al*.^27^. Figure 3(c–h) focus on six Category 5 TCs in the North Atlantic Basin sustaining Category 5 strength for the longest duration in the historical 40-year time period simulated in this dataset. Among these storms, Hurricanes Allen, Irma, Mitch, and Maria reach Category 4 intensity in RAFT’s simulations, while David and Isabel peak at Category 3 intensity. This pattern of under prediction is consistent across other historically simulated Category 5 storms. While RAFT successfully simulates these stronger storms and captures the general trajectory of their intensity changes, it underestimates the peak intensities of the most severe storms. For a more quantitative validation of the dataset, we next evaluate the model’s performance both during training and in full storm simulations. During training, the model’s performance on a validation set of single-step intensity changes from the North Atlantic Basin, derived using the LOYO method, yields a mean error (ME) of -0.22, a mean absolute error (MAE) of 3.44, and a root mean square error (RMSE) of 4.96 (all in knots). When the model is used to autoregressively simulate intensities along entire storm tracks, where errors propagate across time steps, the error increases to an ME of -0.62, an MAE of 14.05, and an RMSE of 20.40 kt. Next, we rerun the historical storms with imposed warming signals to assess how well RAFT’s simulations remain within its valid training envelope. We focus on the SSP585 far-future hot scenario, which produces the largest shifts in environmental inputs. Figure 4 compares each input variable under the historical baseline and the future case: 4(a–d) show variables adjusted by the warming signals, and 4(e–f) display the previous intensity change and current intensity. This analysis confirms that all environmental inputs exhibit only modest distributional shifts and remain within the ranges encountered during RAFT’s historical simulation, avoiding out-of-sample extrapolation.

### RAFT vs WRF-TGW Models and TC Simulations

Before comparing simulated TCs, it’s important to note three key differences in the modeling approaches of RAFT and WRF-TGW. First, RAFT uses deep learning with inputs sourced directly from SHIPS or ERA5, offering an observationally accurate representation of environmental conditions. In contrast, WRF-TGW downscales ERA5 and relies on model physics to simulate TC genesis and TCs. Second, RAFT begins simulating TCs from the first 6-hourly step recorded in the IBTrACS dataset, whereas WRF-TGW tracks storms that form within its domain and begins following those that enter across its boundaries once they meet its detection criteria, potentially missing their earlier development stages. Third, both models follow the same future warming scenarios, but there is a significant difference in how they handle wind in these scenarios. WRF-TGW incorporates primarily temperature and humidity changes in its future signals, along with spectral nudging of winds down to wavenumber three scales. This enables WRF-TGW to incorporate large-scale wind shear directly into the simulations. In contrast, RAFT includes large-scale wind effects as well as vertical wind shear and other smaller-scale wind features derived from the climate model, providing a potentially more nuanced representation of future environmental changes.

Further comparisons with the WRF-TGW approach reveal notable differences in projected TC intensity and variability. For instance, WRF-TGW simulations from both Jones *et al*.^14^ and Zarzycki *et al*.^29^ suggest an average wind speed increase between 1.5 m/s and 57–68% of storms intensifying under future warming scenarios. These dynamical downscaling simulations also show a significant rise in rapid intensification (RI) events, approximately 2.5 times more frequent than observed historically. RAFT, on the other hand, offers a more conservative intensity projection under similar future scenarios, with modest decreases observed under SSP585 far-future conditions. Despite greater intensity variability in dynamical models, RAFT appears to more closely align with observed historical landfall intensities, even as both approaches struggle to replicate the highest Category 5 storm intensities.

**Fig. 5:**
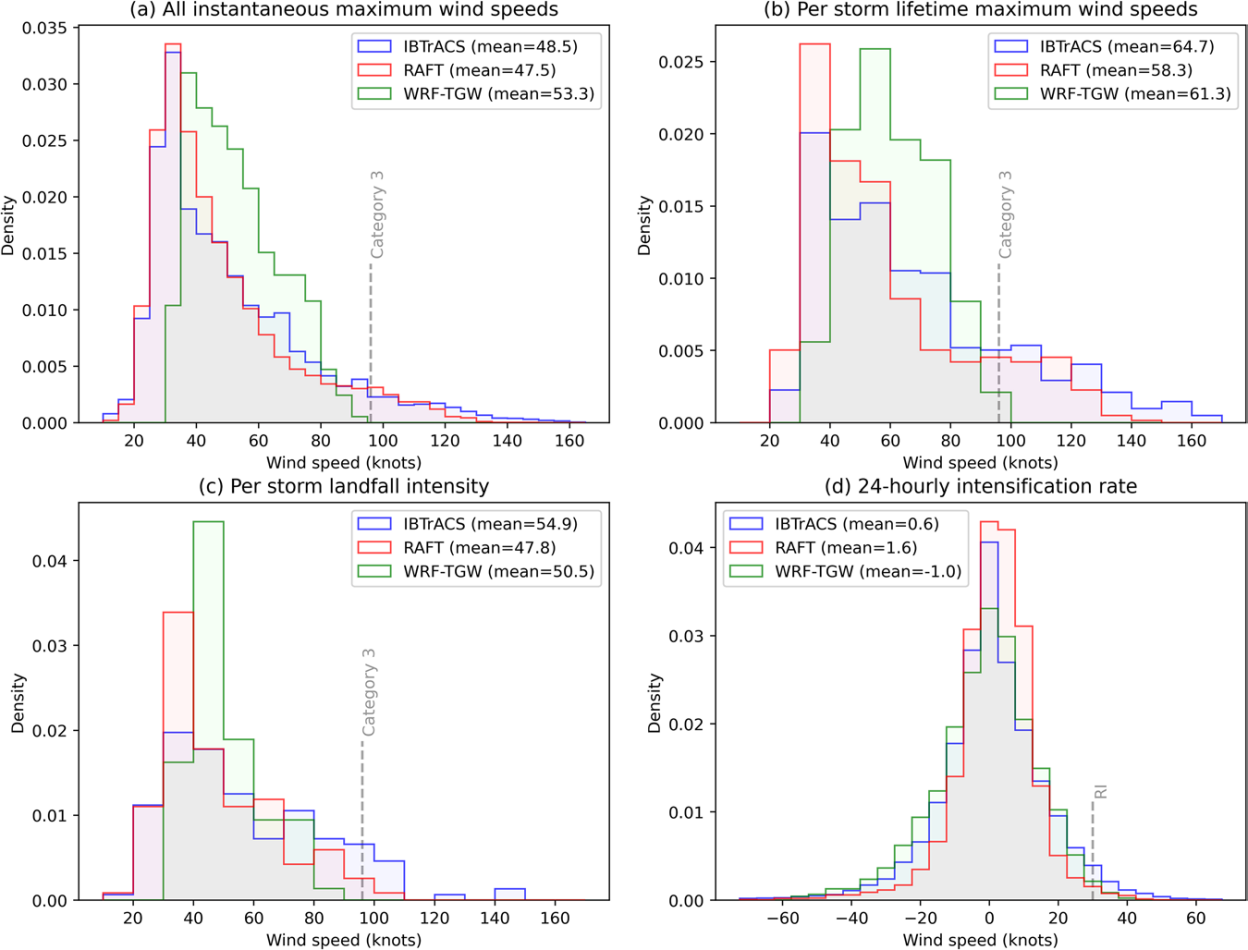
Histograms comparing TC intensity metrics from IBTrACS (blue), RAFT (red), and WRF-TGW (green) for historical simulations. Panels show: (**a**) all instantaneous maximum wind speed, (**b**) per storm lifetime maximum wind speeds, (**c**) per storm landfall intensity over the continental U.S., and (**d**) 24-hourly intensification rate. The y-axis in each panel represents probability density, with the area under each curve summing to 1.

Figure 5(a),(b) illustrate these differences. While WRF-TGW struggles to replicate Category 3 storms and beyond, RAFT successfully simulates stronger storms, albeit with difficulty capturing the intensity maxima of Category 5 storms. Figure 5(c) highlights that both models underestimate storm intensities at landfall, though RAFT’s projections are closer to observed values. Similarly, Fig. 5(d) shows that, while both models acknowledge the importance of RI, neither fully captures the large positive 24-hour intensity changes recorded in IBTrACS, with RAFT tending to overemphasize smaller intensity increases during RI periods.

**Fig. 6:**
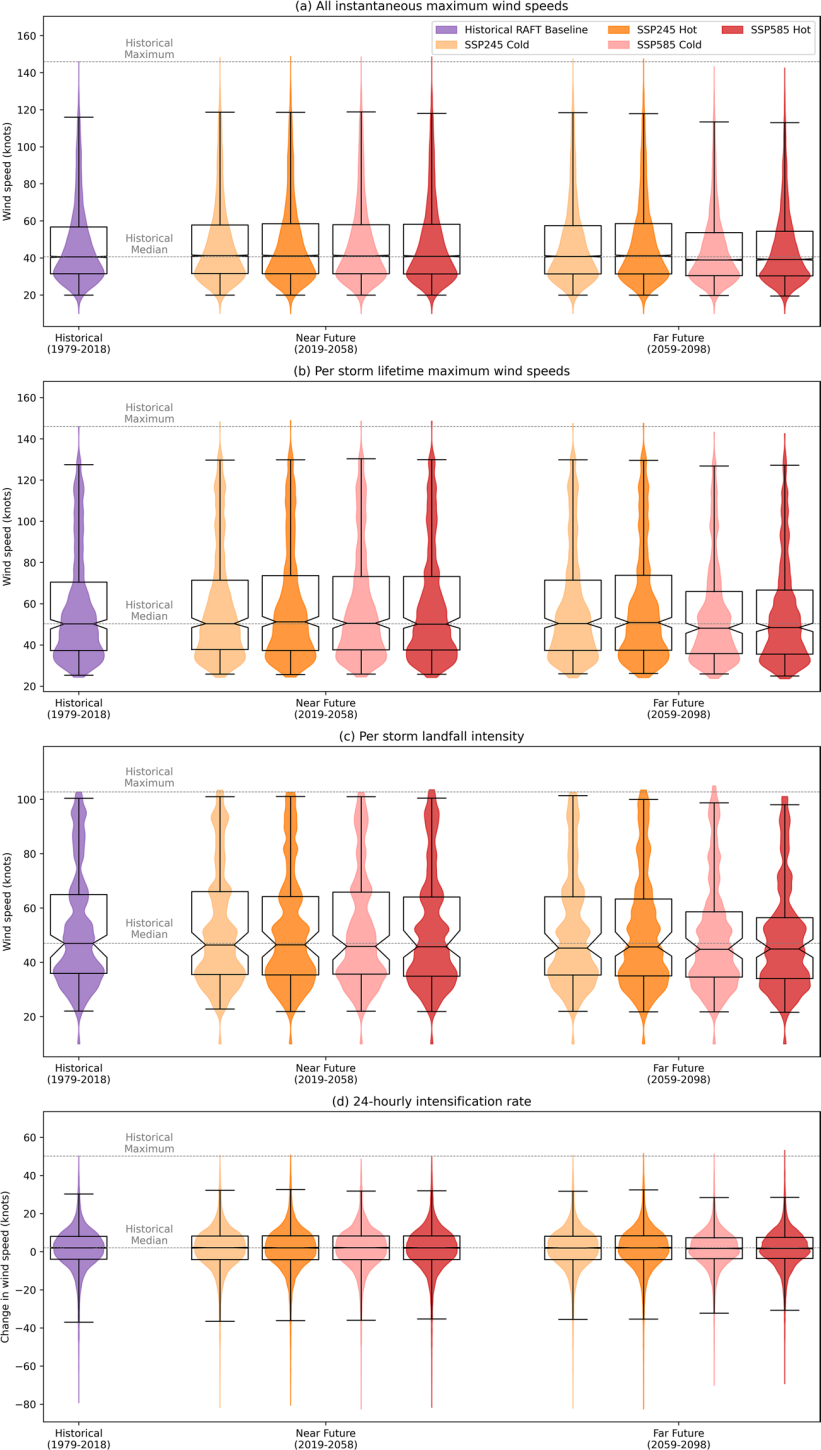
Violin and box-and-whisker plots comparing changes in RAFT’s TC simulations between the historical reproduction and eight future climate scenarios: (**a**) all instantaneous maximum wind speeds, (**b**) per storm lifetime maximum wind speeds, (**c**) per storm landfall intensity over the continental U.S., and (**d**) 24-hourly intensification rate. Boxes represent the 25th, 50th (median), and 75th percentiles, with whiskers indicating the 1st and 99th percentiles. The notch around the median represents the bootstrapped 95% confidence interval of the median.

The differences in TC behavior revealed by these comparisons underscore the advantages and challenges inherent in each approach. RAFT’s deep learning model exhibits greater fidelity in replicating observed TC characteristics, offering a nuanced understanding of TC behavior that complements traditional dynamical approaches like WRF-TGW. Nevertheless, both methods reflect the complexities in projecting TC intensity in a warming climate, highlighting persistent uncertainties and areas for refinement in future studies.

**Fig. 7:**
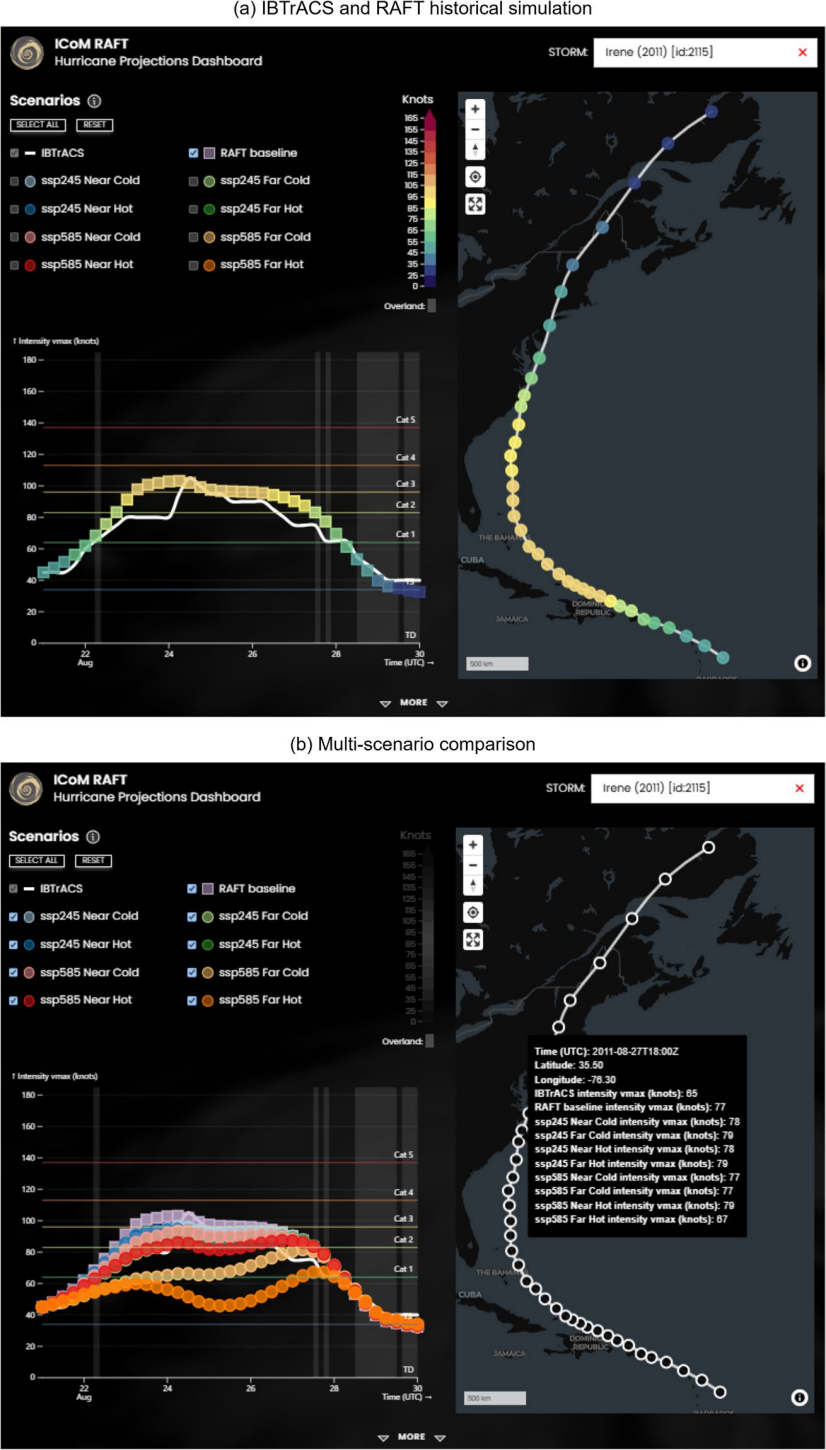
Dashboard for data exploration hosted at RAFT-hurricane-projections.msdlive.org. Panel (**a**) includes the comparison of Hurricane Irene (2011) between RAFT’s historical simulation (multi-colored) and IBTrACS (white), where the map on the right displays RAFT’s simulated intensities. Panel (**b**) shows the comparison of RAFT’s simulation based on eight future climate scenarios against the observed (IBTrACS). When featuring multiple scenarios, hovering the mouse shows information for each data point on the greyed-out map. The difference in simulated landfall intensity between the historical reproduction and the SSP585 far-future hot model shows an increase of roughly 20 knots, escalating landfall intensity from Category 2 to Category 3.

## Usage Notes

### Limitations and Assumptions of the Model

A comprehensive understanding of the assumptions and limitations of this study which uses the deep learning intensity model in RAFT is essential for effectively interpreting its results. This storyline experiment uses fixed initial conditions and storm tracks across all scenarios, which means it does not account for environmental dynamics that could influence TC genesis, track shifts, translation speed, or SST changes due to cool wake effect^30,31^. Additionally, the training data is imbalanced, with an over-representation of over-ocean storm steps and a limited number of Category 5 TCs. This imbalance contributes to the model’s difficulty in accurately simulating landfall intensities and peak intensities of extreme events. With only 10 input variables, the model may miss critical environmental factors, which might account for the less accurate simulations of some historical TCs and the very poor simulations of certain storms. Users should critically assess the accuracy of historical simulations for specific storms before drawing conclusions from future projections.

**Fig. 8:**
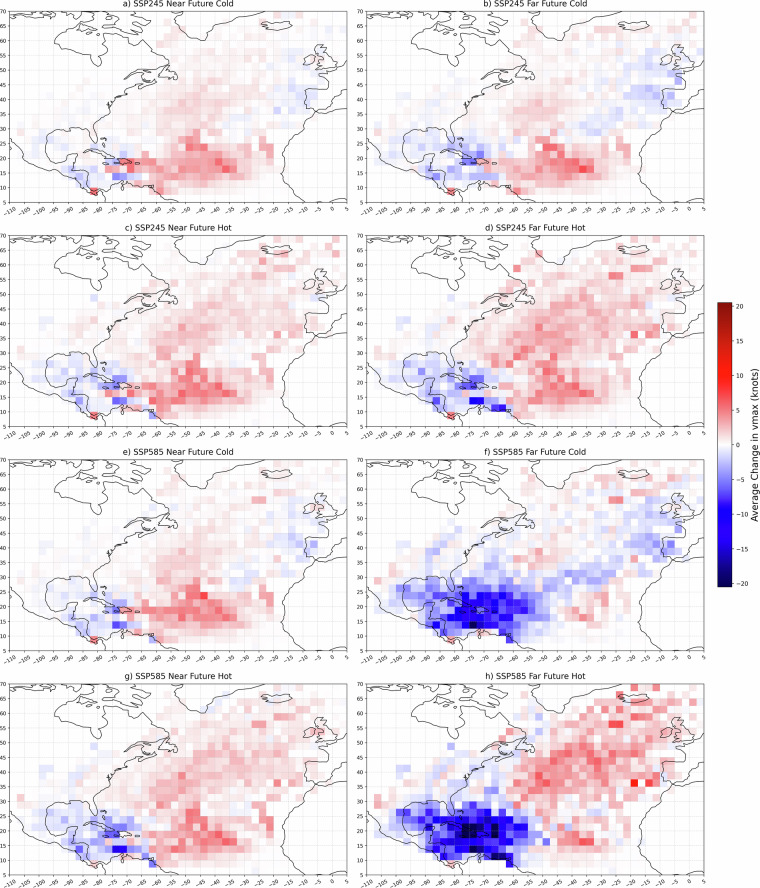
Average change in intensity between historical baseline and future simulations on a 2.5 degree grid.

## Data Availability

The dataset generated in this study is openly available at 10.57931/2588708^28^.

## Code availability

Code to read and process the dataset can be found at 10.57931/2588708^28^.
